# Risk Factors for Failure of Second-Trimester Termination with Misoprostol as a Single Agent

**DOI:** 10.3390/jcm13175332

**Published:** 2024-09-09

**Authors:** Veera Wisanumahimachai, Saipin Pongsatha, Latchee Chatchawarat, Theera Tongsong

**Affiliations:** Department of Obstetrics and Gynecology, Faculty of Medicine, Chiang Mai University, Chiang Mai 50200, Thailand; wira.w@cmu.ac.th (V.W.); latchee.chat@cmu.ac.th (L.C.)

**Keywords:** abortion, failure of pregnancy termination, mid-pregnancy, misoprostol, risk factor, second trimester

## Abstract

**Background:** Understanding the potential risk factors for failure of pregnancy termination is crucial for informed clinical decision making. Such insights can assist clinicians in adjusting the dosage or route of various regimens, as well as in counseling patients and predicting the likelihood of successful outcomes. However, research on these risk factors has been limited, and existing studies have yielded inconsistent results. To address this gap, we conducted a study with a large sample size, focusing on identifying the potential risk factors for failure of second-trimester termination using misoprostol as a single agent, specifically between 14 and 28 weeks of gestation. **Methods:** A secondary analysis based on a database of second-trimester terminations was conducted. The inclusion criteria were a singleton pregnancy, gestational age between 14 and 28 weeks, an unfavorable cervix, no spontaneous labor pain, intact membranes, and termination with misoprostol alone. Potential risk factors for failure of termination, defined as no abortion within 48 h, were analyzed using univariate and multivariate analyses. **Results:** A total of 1094 cases were included in the analysis, consisting of 991 successful cases and 103 (9.4%) cases of failure. The significant risk factors for failure of termination included early gestational age, live fetuses, sublingual regimen of 400 mcg every 6 h, and high maternal pre-pregnancy BMI. Previous cesarean sections and lower Bishop scores tended to increase the risk but did not reach a significant level. **Conclusions:** Second-trimester termination with misoprostol as a single agent was highly effective, with a failure rate of 9.4%. The risk factors for failure included gestational age, fetal viability, misoprostol regimen, and maternal pre-pregnancy BMI, suggesting that these factors should be taken into consideration for second-trimester terminations with misoprostol.

## 1. Introduction

Second-trimester pregnancy termination presents significant challenges. Several methods, both surgical and medical, have been proposed and studied. A surgical method involving dilation, evacuation, and curettage has been performed in some institutions with a high success rate and is usually completed quickly [1,2,3,4]. However, this procedure is invasive and carries a theoretical risk of massive bleeding, especially in less experienced hands, due to the larger size of the uterus compared to the first trimester. This procedure also typically requires anesthesia, making it suitable only for experienced practitioners in settings with high safety standards, including access to an operating room, anesthesiologists, and blood transfusion services. As a result, surgical evacuation is not commonly performed worldwide. Mechanical approaches [5,6,7,8,9,10,11,12], such as the use of a hydrostatic catheter balloon to separate the membranes of the lower uterine segment or the insertion of a laminaria tent to facilitate cervical dilation and softening, have also been employed, albeit with varying success rates. Despite being minimally invasive, these methods are not widely available and lack extensive data on their efficacy, limiting their widespread use. Medical management of second-trimester termination has been extensively researched. Prostaglandins, particularly misoprostol (prostaglandin E1 analogue) and prostaglandin E2, are the primary medications used for this purpose. Misoprostol, in particular, has become the most commonly used drug due to its stability at room temperature, low cost, wide availability, and high efficacy. Various regimens involving misoprostol have been studied, including different routes of administration (intravaginal, intracervical, oral, or sublingual), dosages, intervals between doses, and use as a single agent or in combination with other medications, especially mifepristone, or with mechanical methods. Currently, the combination of misoprostol and mifepristone is considered the preferred method, as supported by several studies and meta-analyses [13,14,15,16,17,18,19,20,21] and recommendations from major health organizations [22,23]. However, due to the unavailability and higher cost of mifepristone, misoprostol alone remains the most commonly used method worldwide, and is recommended as a reasonable alternative approach, especially in low-resource settings and developing countries where mifepristone is not available. In addition to high efficacy, misoprostol has advantages such as low cost, worldwide availability, and stability in room air, making it simple to handle and preserve, while the side effects and complications are acceptable. Therefore, misoprostol is the most commonly used medication worldwide [24].

Knowledge concerning potential risk factors for failure is important for clinical decision making in management. Such knowledge can help clinicians adjust dosages or routes of different regimens and is also useful for patient counseling and predicting successful outcomes. Fetal life status, gestational age, misoprostol regimens, and maternal body mass index (BMI) have been reported to affect the success rate of termination [25,26,27,28]. Nevertheless, the number of such studies is very limited and mostly based on small sample sizes or heterogeneous data without control of confounding factors. This might be due to the fact that misoprostol is highly effective in pregnancy termination, resulting in only a small number of cases with termination failure in most previous studies, leading to difficulty in analyzing the risk factors for failure with high reliability. Additionally, some potential risk factors may theoretically increase the risk of failure, but no confirmatory studies support this. For example, parity might affect the failure rate of second-trimester termination, as there is evidence that high parity is an independent factor associated with a higher success rate in labor induction at term than low parity [29]. It is well known that multiparity shortens the labor curve compared to nulliparity. A prolonged labor duration could extend the overall induction time beyond 48 h, meeting the criteria for induction failure. Therefore, theoretically, parity might influence the outcome of second-trimester termination as well. However, no conclusive evidence has been established in the literature regarding whether parity is associated with the failure rate of second-trimester termination when using misoprostol. Therefore, we conducted this study with a large sample size, primarily aimed at determining the potential risk factors for failure of second-trimester termination with misoprostol as a single agent between 14 and 28 weeks of gestation.

## 2. Patients and Methods

A retrospective study as a secondary analysis based on the prospective database of pregnancy termination in the second trimester was conducted at Maharaj Nakorn Chiang Mai Hospital, a tertiary center and university hospital at Chiang Mai University, Thailand, between June 1997 and December 2023. The study was ethically approved by the Institutional Review Boards (Research Ethics Committee 4, Faculty of Medicine, Chiang Mai University; study code ID: OBG-2566-09390, approval date 11 May 2023).

The database of the misoprostol study was first created in 1997 to collect all consecutive cases of misoprostol use in our department, involving several projects with various regimens based on the active project at different study periods. Upon database development, written informed consent was provided by the patients. The main data included baseline characteristics (such as maternal age, parity, obstetric history, and gestational age) and clinical data (such as indications for pregnancy termination, Bishop score, misoprostol regimens, natural course of labor and abortion, side effects of misoprostol, requirement of oxytocin and analgesia, induction-to-abortion time, total misoprostol doses used, and estimated blood loss), which were assessed and prospectively recorded in the research forms.

The data used in this analysis were retrieved from the database and a comprehensive review of the full medical records. The women meeting the following inclusion criteria were included in this analysis: (1) singleton gestation; (2) gestational age of 14 to 28 weeks; (3) having an indication for termination of pregnancy; (4) no cervical ripening (Bishop score ≤ 4); (5) absence of labor pain and rupture of membranes; and (6) undergoing pregnancy termination with misoprostol as a single agent, with the exception that oxytocin might be added after successful cervical ripening to adjust uterine contractions as appropriate. The exclusion criteria were as follows: (1) twin pregnancies; (2) rupture of membranes; (3) placenta previa; (4) allergy to misoprostol; (5) regimens with a small sample size (fewer than 50 cases); and (6) incomplete data.

The main outcome measure was failure of termination, defined as no abortion (fetal delivery) within 48 h after initiation of misoprostol administration. The potential risk factors considered included maternal age, parity, pre-pregnancy body mass index (BMI: kg/m^2^), fetal viability (a dead or live fetus), gestational age at termination, misoprostol regimens, Bishop scores, and history of previous cesarean section.

All patients were typically cared for under the same standard protocol as follows: After starting the first dose, misoprostol was repeated at scheduled times depending on the regimen in cases of persistent unfavorable cervix or no achievement of adequate uterine contractions. If adequate uterine contractions were achieved, though the cervix was unfavorable, the scheduled dose was skipped and reevaluated again at the scheduled time, and misoprostol was then resumed if uterine contraction was still inadequate and the cervix was still unfavorable. If the cervix became favorable but uterine contractions were inadequate, misoprostol was discontinued, and intravenous oxytocin was then infused by automatic infusion pump, with dose adjustment as appropriate. Intravenous meperidine (50 mg) for painful labor was given upon patient request. Note that, in second-trimester termination, according to the standard practice at our institution, we used misoprostol alone and did not use intracardiac potassium injections for viable fetuses, even in cases beyond 22 weeks of gestation.

### Statistical Analysis

In statistical comparisons, the Chi-square test was used for categorical data, and the Student’s *t*-Test or Mann–Whitney U test was used for continuous data as appropriate. Binary logistic regression was used for univariate and multivariate analyses to assess the potential risk of various factors for termination failure. Kaplan–Meier curves with a log-rank test were applied to compare the effectiveness of various groups of regimens in inducing termination. All statistical procedures were performed using the Statistical Package for the Social Sciences (SPSS) software version 26.0 (IBM Corp. Released 2019. IBM SPSS Statistics for Windows, Armonk, NY, USA: IBM Corp). A *p*-value of less than 0.05 was considered statistically significant.

## 3. Results

**Figure 1:**
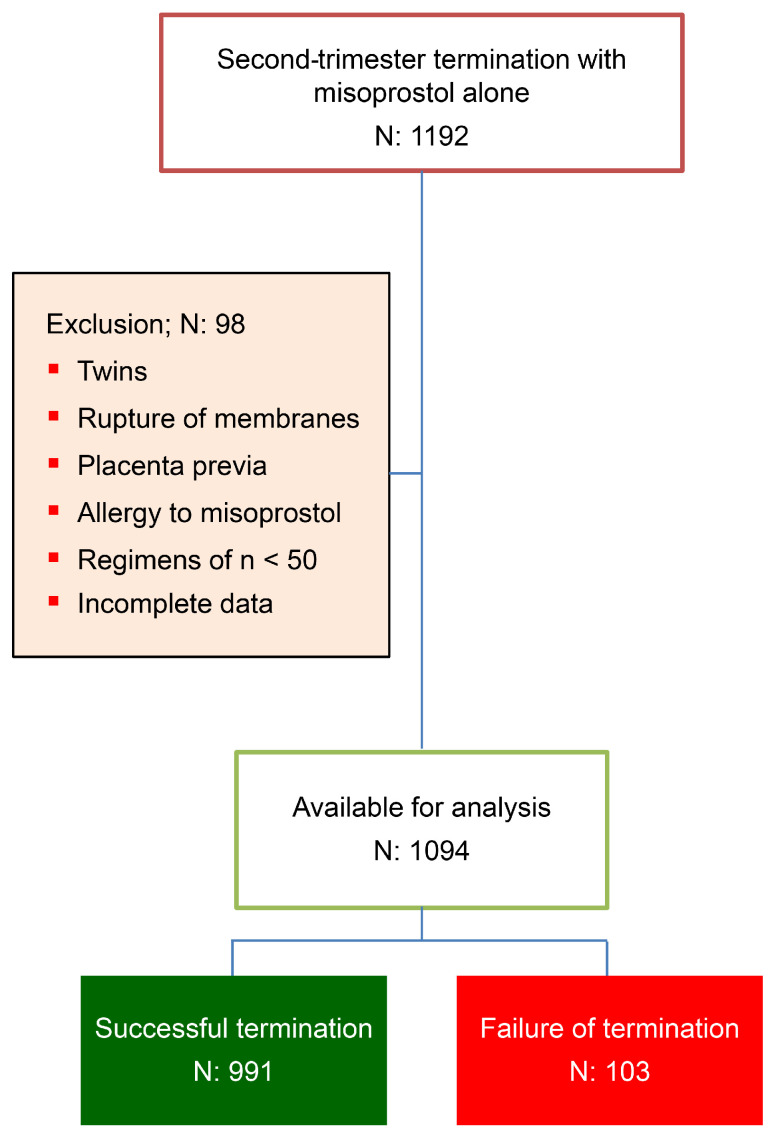
Flowchart of patient recruitment.

During the study period, a total of 1192 pregnancies underwent second-trimester termination with misoprostol as a single agent. Of these, 98 were excluded for various reasons, as shown in Figure 1. The remaining 1094 cases were included in the analysis, comprising 991 cases (90.6%) with successful termination within 48 h and 103 cases (9.4%) with failure. Comparisons of baseline data between the success group and the failure group revealed that most baseline characteristics were not significantly different, as presented in Table 1. However, a significant difference was observed in the mean gestational age of pregnancy termination (21.6 ± 2.7 weeks vs. 20.2 ± 3.0 weeks, *p* < 0.001), indications for termination, and misoprostol regimens used. The most common indication for termination was severe fetal thalassemia, followed by fetal structural anomalies and fetal chromosomal/structural abnormalities, while intrauterine fetal death accounted for approximately one-fifth of cases. The two most common misoprostol regimens were vaginal 400 mcg every 6 h, accounting for nearly half of the cases, and vaginal 400 mcg every 3 h, accounting for about one-third.

In determining the potential risk factors for termination failure, the univariate analysis showed that advancing gestational age and fetal death were significantly associated with lower risks for failure of termination, while other potential risk factors were not significantly associated with the failure rate, as presented in Table 2. Overall, the misoprostol regimens had comparable failure rates, but a subgroup analysis indicated that sublingual 400 mcg every 6 h significantly increased the failure rate compared to the vaginal 400 mcg every 6 h regimen. On multivariate analysis, gestational age and fetal death were still significantly associated with the failure rate. Notably, the regimens also significantly affected the failure rate, with sublingual 400 mcg every 6 h still showing a significant increase in the failure rate. Interestingly, pre-pregnancy BMI, which was not significantly associated with the failure rate in the univariate analysis, significantly increased the risk of failure after controlling for other factors.

**Figure 2:**
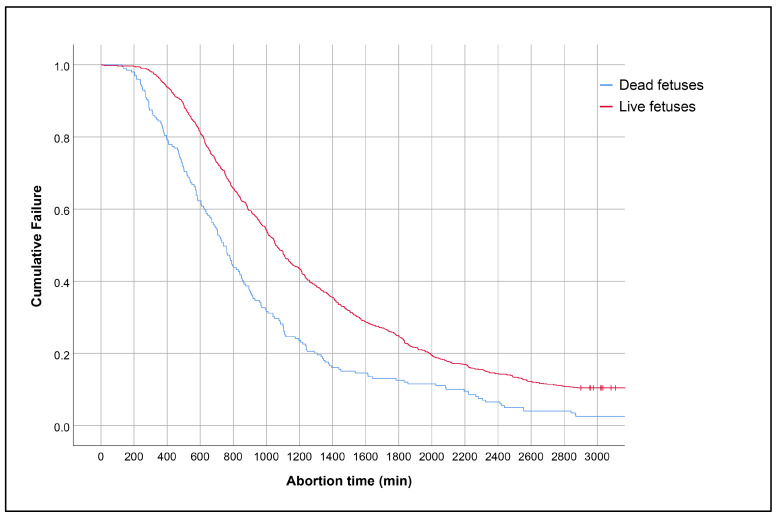
Kaplan–Meier curves of abortion time duration based on fetal viability (log-rank test, *p* < 0.001).

In addition to the observation that fetal death had a strong effect on decreasing the risk of failure, with an odds ratio of about 0.1, the Kaplan–Meier curve of abortion time in the cases with dead fetuses was significantly shorter than those with live fetuses (log-rank test, *p* < 0.001), as presented in Figure 2.

**Figure 3:**
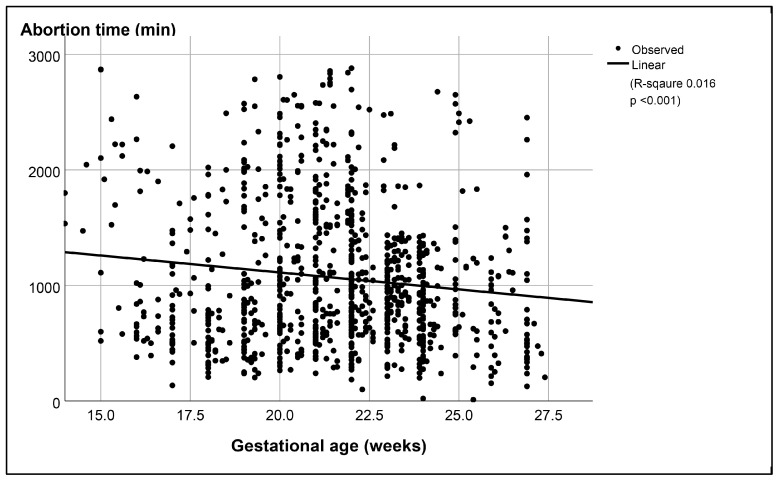
Significant correlation between gestational age and abortion time among cases of success.

**Figure 4:**
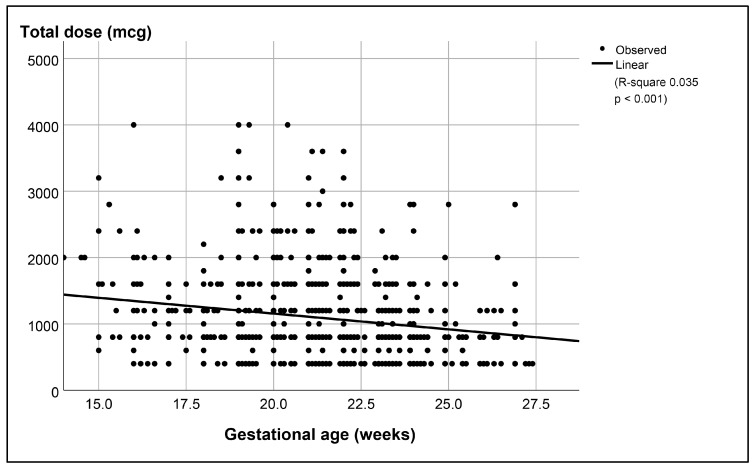
Significant correlation between gestational age and total dose of misoprostol used among cases of success.

In the overall regression analysis, gestational age was also significantly associated with abortion time (*p* < 0.001), as presented in Figure 3, and was significantly associated with the total doses of misoprostol required for successful termination (*p* < 0.001), as presented in Figure 4. In a subgroup analysis that compared the failure rate between the subgroup with a gestational age of 22 weeks or more and the subgroup with a gestational age of less than 22 weeks, the subgroup with a gestational age of less than 22 weeks had a significantly higher failure rate (*p*-value 0.001, with a relative risk of 1.90), as presented in Table 3.

**Figure 5:**
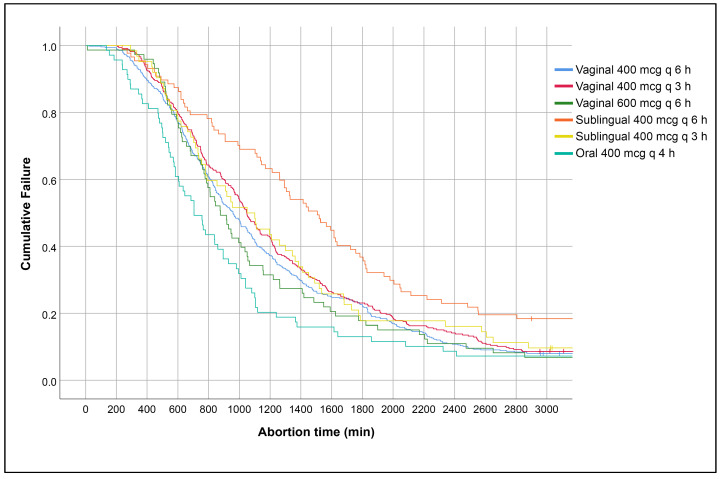
Kaplan–Meier curves of abortion time duration based on misoprostol regimens (log-rank test, *p* < 0.001).

Among the various regimens, the median time for success based on Kaplan–Meier curves and the mean duration of abortion time among the successful cases were significantly higher in the group receiving sublingual 400 mcg every 6 h and significantly lower in the group receiving oral 400 mcg every 4 h, whereas the other regimens were comparable, as presented in Table 4 and Figure 5.

## 4. Discussion

The insights gained from this study are as follows: (1) The overall failure rate of misoprostol used as a single agent in second-trimester termination is approximately 9%. (2) The significant risk factors for increased failure rate are earlier gestational age, pregnancies with a live fetus, high maternal pre-pregnancy BMI, and the sublingual regimen of 400 mcg every 6 h. Notably, other potential risk factors like low Bishop scores and previous cesarean sections tended to increase the failure rate but did not reach a significant level, possibly due to the low power of the test to detect a significant effect if it existed. (3) Although most regimens were comparable in terms of failure rates, the oral regimen of 600 mcg every 4 h yielded the lowest failure rate. Additionally, according to the Kaplan–Meier curve, vaginal 600 mcg every 6 h seemed to be superior to the 400 mcg regimen (shorter abortion time), although they had the same failure rate at 48 h. Nevertheless, in terms of failure rate within 48 h, all regimens, except sublingual 400 mcg every 6 h, were comparable in effectiveness. Therefore, this study suggests that the regimen of vaginal 400 mcg every 6 h may be the most preferred because of its lower dosage and longer interval of administration, which are likely associated with fewer side effects and complications. As a safety concern for widespread use, especially in low-resource settings or where there is a limited number of caregivers, the low dosage and long interval are preferred.

Our findings suggest that gestational age is a strong independent factor for termination failure. However, the evidence in the literature supporting this finding is limited. Consequently, some guidelines for second-trimester termination do not take gestational age into account. For example, WHO guidelines [23] recommend misoprostol 400 mcg every three hours for pregnancy termination at gestational ages between 12 and 28 weeks. Nevertheless, our study supports the FIGO guideline [22], which recommends different misoprostol dosages for different gestational ages: 400 mcg every three hours for 13–24 weeks and 200 mcg every four hours for 25–28 weeks.

Several guidelines recommend the same regimens regardless of fetal life status. However, our findings indicate that the presence of a live fetus was also an independent risk factor for termination failure, consistent with observations in some previous studies [28]. Therefore, appropriate guidelines should take the status of fetal life into consideration. Our results support the 2017 FIGO guideline [22], which recommends a lower dosage of misoprostol for pregnancies with fetal death and a higher dosage for those with a live fetus. However, we have observed that in 2023, the FIGO website published the ‘Misoprostol Only Dosing Charts 2023’ [30], which recommends the same misoprostol dosage for pregnancies with both fetal death and a live fetus. These inconsistent recommendations by FIGO are likely based on limited evidence and expert opinion. Based on our findings, using the same regimen for both deceased and live fetuses at all gestational ages during the second trimester, as recommended by many guidelines, including our own practice, is no longer justified.

Notably, BMI had a significant effect on increasing the failure rate. This finding is consistent with some previous studies [25,29,31], which demonstrated that increased BMI or overweight/obesity was significantly associated with less successful induction of labor by misoprostol. However, the effect of BMI in our study was minimal, though significant, and was not evident in univariate analysis, possibly due to it being masked or confounded by other risk factors. Therefore, the effect of BMI might not be clinically significant.

The strengths of this study are as follows: (1) a large sample size; (2) although it is a retrospective study, the database was prospectively collected and comprehensively reviewed at the time of admission; and (3) high homogeneity of medication, with misoprostol used as a single agent.

The limitations of this study are as follows: (1) the reliability of the conclusions might not be perfect due to its retrospective nature, although it is based on a prospective database; (2) although the overall sample size was relatively large, the number of cases for some misoprostol regimens was rather low; (3) the long time frame of the study, with changes in clinical care over the study period, which might affect the outcome; and (4) some other regimens of misoprostol (with small sample sizes) or in combination with other methods were excluded.

Research implications: Future studies should incorporate all potential risk factors to develop a predictive model, particularly by using machine learning techniques, to estimate the failure rate of termination. This model may help guide the selection of the most appropriate regimen for individual patients, rather than applying a single regimen universally. Importantly, the data from this study could serve as a valuable resource for future systematic reviews or meta-analyses on the effectiveness of misoprostol as a single agent. Finally, comparisons should be made between regimens recommended by major health organizations, such as FIGO or WHO, especially when used either as a single agent or in combination with other medications like mifepristone.

## 5. Conclusions

This study provides evidence that may be useful for patient counseling and prediction of failed termination. Importantly, this study suggests that in the clinical practice of second-trimester termination, gestational age and fetal life status should be taken into consideration when selecting misoprostol regimens, which might help lower the failure rate or shorten abortion time.

## Figures and Tables

**Table 1:** Baseline characteristics of the patients.

Characteristics	Success (n = 991)	Failure (n = 103)	*p*-Value
Age (years)	30.77 ± 6.86	30.29 ± 7.33	0.503 (a)
Body mass index, mean ± SD	20.41 ± 2.40	20.54 ± 2.80	0.585 (a)
Gestational age (weeks), mean ± SD	21.6 ± 2.7	20.2 ± 3.0	<0.001 (a)
Birth weight	403 ± 256	348 ± 265	0.039 (a)
Initial Bishop score, mean ± SD	1.47 ± 0.99	1.50 ± 0.96	0.774 (a)
Parity, n (%)			0.061 (b)
• 0	514 (51.9%)	59 (57.3%)	0.295
• 1	355 (35.9%)	34 (33.0%)	0.570
• 2	91 (9.2%)	9 (8.7%)	0.882
• 3	24 (2.4%)	0 (0.0%)	0.112
• 5 or more	4 (0.4%)	1 (1.0%)	0.417
Indication for pregnancy termination, n (%)			0.006 (b)
• Intrauterine fetal death	194 (19.6%)	5 (4.9%)	<0.001
• Fetal structural anomalies	279 (28.2%)	35 (34.0%)	0.213
• Fetal chromosomal abnormalities	163 (16.4%)	17 (16.5%)	1.000
• Fetal severe thalassemia	291 (29.4%)	39 (37.9%)	0.074
• Others	26 (2.6%)	1 (1.0%)	0.304
Previous cesarean section	85 (9.1%)	13 (12.6%)	0.246
Misoprostol regimen, n (%)			0.042 (b)
• Vaginal 400 mcg q 6 h	435 (43.9%)	42 (40.8%)	0.543
• Vaginal 400 mcg q 3 h	297 (30.0%)	28 (27.2%)	0.556
• Vaginal 600 mcg q 6 h	68 (6.9%)	5 (4.9%)	0.437
• Sublingual 400 mcg q 6 h	71 (7.2%)	17 (16.5%)	0.001
• Sublingual 400 mcg q 3 h	56 (5.7%)	6 (5.8%)	0.944
• Oral 400 mcg q 4 h	64 (6.5%)	5 (4.9%)	0.524

(a) Student’s *t*-Test; (b) Chi-square test.

**Table 2:** Univariate and multivariate analyses of potential risk factors for termination failure.

	Univariate Analysis		Multivariate Analysis	
	*p*-Value	Odds Ratio (95%CI)	*p*-Value	Odds Ratio (95%CI)
Maternal age	0.502	0.99 (0.96–1.02)	0.630	0.99 (0.96–1.03)
Parity	0.300	1.24 (0.82–1.87)	0.219	1.37 (0.83–2.27)
Gestational age	<0.001	0.84 (0.78–0.92)	<0.001	0.84 (0.77–0.91)
Fetal death	0.001	0.21 (0.08–0.52)	0.001	0.09 (0.02–0.37)
Regimens	0.055		0.017	-
▪ Vaginal 400 mcg q 6 h	Ref	Ref	Ref	Ref
▪ Vaginal 400 mcg q 3 h	0.926	0.98 (0.59–1.61)	0.386	0.79 (0.46–1.35)
▪ Vaginal 600 mcg q 6 h	0.579	0.76 (0.29–1.99)	0.376	0.64 (0.24–1.72)
▪ Sublingual 400 mcg q 6 h	0.004	2.48 (1.34–4.60)	0.012	2.32 (1.21–4.44)
▪ Sublingual 400 mcg q 3 h	0.821	1.11 (0.45–2.73)	0.717	0.84 (0.33–2.15)
▪ Oral 400 mcg q 4 h	0.667	0.81 (0.31–2.12)	0.088	3.72 (0.82–16.80)
Previous cesarean section	0.249	1.44 (0.77–2.69)	0.288	1.52 (0.70–3.31)
Bishop scores (0–4)	0.066	0.82 (0.67–1.01)	0.433	0.91 (0.73–1.14)
Body mass index (kg/m^2^)	0.584	1.02 (0.94–1.11)	0.006	1.16 (1.04–1.28)

**Table 3:** Comparison of failure rate between subgroup with gestational age of less than 22 weeks and subgroup with gestational age of 22 weeks or greater.

Gestational Age Group	No Failure	Failure	*p*-Value * and Relative Risk (95%CI)
Less than 22 weeks	496 (87.8%)	69 (12.2%)	0.001 1.90 (1.28–2.81)
22 weeks or more	495 (93.6%)	34 (6.4%)	
Total	991 (90.6%)	103 (9.4%)	

* Chi-square test.

**Table 4:** Abortion time based on regimens.

	Median Time for Success (Based on Kaplan Meier Curve) (Minutes) *	Mean Duration (±SD) (Minutes) among Successful Cases #
Vaginal 400 mcg q 6 h	959 (880–1038)	1044 ± 601
Vaginal 400 mcg q 3 h	1048 (976–1120)	1118 ± 622
Vaginal 600 mcg q 6 h	877 (750–1004)	1014 ± 588
Sublingual 400 mcg q 6 h	1508 (1237–1779)	1280 ± 634
Sublingual 400 mcg q 3 h	1050 (775–1325)	1102 ± 632
Oral 400 mcg q 4 h	705 (565–845)	798 ± 497

* Log-rank test, *p* < 0.001; # ANOVA test, *p* < 0.001.

## Data Availability

The datasets analyzed during the current study are available from the corresponding author upon reasonable request.
